# Transcriptome Analysis of Porcine Granulosa Cells in Healthy and Atretic Follicles: Role of Steroidogenesis and Oxidative Stress

**DOI:** 10.3390/antiox10010022

**Published:** 2020-12-28

**Authors:** Li Meng, Zhenfang Wu, Kun Zhao, Jian Tao, Tam Chit, Shouquan Zhang, Chi Chiu Wang, Katja Teerds

**Affiliations:** 1 National Engineering Research Center of Breeding Swine Industry, College of Animal Science, South China Agricultural University, Guangzhou 510642, China; limeng@scau.edu.cn (L.M.); zhao971111@gmail.com (K.Z.); taojian19922020@gmail.com (J.T.); 2 Guangdong Provincial Key Lab of Agro-Animal Genomics and Molecular Breeding, Key Lab of Chicken Genetics, Breeding and Reproduction, Ministry of Agriculture, South China Agricultural University, Guangzhou 510642, China; 3 Department of Obstetrics & Gynaecology, Li Ka Shing Institute of Health Sciences, School of Biomedical Sciences, The Chinese University of Hong Kong, Shatin, Hong Kong; kentamc@link.cuhk.edu.hk (T.C.); ccwang@cuhk.edu.hk (C.C.W.); 4 Human and Animal Physiology, Wageningen University, De Elst 1, 6708 WD Wageningen, The Netherlands; katja.teerds@wur.nl

**Keywords:** transcriptome profiles, steroidogenesis, oxidative stress, antral follicular atresia

## Abstract

One of the main causes of female infertility is a deregulated antral follicular atresia, a process of which the underlying molecular mechanisms are largely unknown. Our objective was therefore to characterize the complex transcriptome changes in porcine granulosa cells of healthy antral (HA) and advanced antral atretic (AA) follicles, using ELISA and RNA-Seq followed by qRT-PCR and immunohistochemistry. Granulosa cell RNA-Seq data revealed 2160 differentially expressed genes, 1483 with higher and 677 with lower mRNA concentrations in AA follicles. Bioinformatic analysis showed that the upregulated genes in AA follicles were highly enriched in inflammation and apoptosis processes, while the downregulated transcripts were mainly highlighted in the steroid biosynthesis pathway and response to oxidative stress processes including antioxidant genes (e.g., *GSTA1*, *GCLC*, *GCLM*, *IDH1*, *GPX8*) involved in the glutathione metabolism pathway and other redox-related genes (e.g., *RRM2B*, *NDUFS4*). These observations were confirmed by RT-qPCR and immunohistochemistry. Additionally, the granulosa cells of AA follicles express significantly stronger 8-OHdG immunostaining, a marker of oxidative DNA damage, implicating that oxidative stress may participate in follicular atresia. We hypothesize that the decrease in anti-apoptotic factors and steroid hormones coincides with increased oxidative stress markers and the expression of pro-apoptotic factors, all contributing to antral follicular atresia.

## 1. Introduction

The main function of the ovary is the production of steroid hormones necessary for the development of female secondary sexual characteristics, and to provide an optimal environment for oocyte maturation and release [1,2]. Ovarian follicular development is a process regulated by various factors including gonadotropins and growth factors all involved in the process of selecting the best follicles for ovulation [3]. The vast majority of follicles, however, (between 90–99% of follicles, depending on the species) fail to reach the preovulatory stage, but instead undergo degeneration by a process named atresia [2]. Although atresia is considered a physiological process ensuring that only oocytes of the best quality from the healthiest follicles will ovulate, dysregulated atresia can lead to infertility and premature menopause in women at a reproductive age [3]. 

Nowadays, it is generally accepted that antral follicular atresia is initiated when granulosa cells fail to receive sufficient signals to suppress the apoptotic pathway and/or when pro-apoptotic factors are released, promoting granulosa cell apoptosis [4]. Examples of anti-apoptotic factors include gonadotropins, estrogens, and growth factors like insulin-like growth factor 1 (IGF1) [5]. Androgens produced by theca interna cells are, once arrived in the granulosa cells, converted into β-estradiol by the enzyme aromatase, a process in the porcine ovary under the control of FSH and LH. Estradiol influences various process within the ovary such as folliculogenesis thereby inhibiting granulosa cell apoptosis. Our previous study [6] and Tilly et al. [7] both showed that estradiol concentrations in follicular fluid of atretic antral follicles are significantly lower than those in heathy follicles. Moreover, estradiol and progesterone can improve porcine and bovine oocyte developmental competence during in vitro maturation, when supplemented at the optimal time and dosage [8]. Costermans et al. reported that β-estradiol levels are positively associated with follicle size and not surprisingly sows with a high percentage of healthy COCs have higher follicular fluid levels of β-estradiol and progesterone [9]. Although studies have emphasized the association between steroid hormone profiles and follicle development, the molecular mechanisms underlying the way steroidogenesis affects follicular atresia are less well defined.

Oxidative stress, induced by an imbalance between reactive oxygen species (ROS) production and cellular antioxidant defense capacity, has long been hypothesized to be one of the factors capable of triggering apoptosis and thus follicular atresia, especially under the presence of stressors such as aging [10,11] and hypothyroidism [12]. Additionally, it is speculated that estrogens may exert an anti-atretic effect by modulation of intrafollicular oxidative stress [4]. Support for this assumption comes from several, mainly in vitro, studies. For example, it is demonstrated that granulosa cells isolated from ovine and porcine large antral follicles, are protected from oxidative (H_2_O_2_) stress-induced apoptosis when the culture medium is supplemented with estradiol [13,14]. Direct in vivo evidence for a physiological role of oxidative stress in antral follicular atresia is still largely lacking. The few studies on molecular changes during follicular atresia used microarrays to investigate gene expression and mainly focused on either small antral follicles (<2 mm in diameter) [15] or early stages of antral follicular atresia [16].

The aim of the present study is to analyze granulosa cells from medium to large-sized (4–7 mm in diameter) healthy antral (HA) and advanced atretic antral (AA) follicles using next generation RNA sequencing (RNA-seq) to obtain a better understanding of global transcriptome-wide gene expression profiles associated with follicle atresia, and to identify novel potential molecular biomarkers for atretic follicles. In this study, HA follicles and advanced AA follicles (4–7 mm in diameter) were dissected and granulosa cells collected for RNA-seq, followed by pathway analysis, qRT-PCR, and immunohistochemical staining to confirm gene expression data.

## 2. Materials and Methods 

### 2.1. Chemicals 

All chemicals were purchased from Sigma (Guangzhou, China) unless indicated otherwise. Antibodies against glutamate-cysteine ligase catalytic subunit (GCLC, lot no. GR3252900-3, cat. no. ab131442), Ki67 (lot no. GR3198158-1, cat. no. ab15580) and aromatase (lot no. GR3231482-1, cat.no. ab18995) were purchased from Abcam (Cambridge, UK). The antibody against 8-hydroxydeoxyguanosine (8-OHdG, lot no L2019, cat. no. sc66036) was purchased from Santa Cruz (Santa Cruz Biotechnology, Hongkong).

### 2.2. Animal and Follicle Collection

The granulosa cells used in this study were collected during a previous experiment [6]. In brief, 60 porcine ovaries from 30 gilts (nulliparous, around 180 days old with a bodyweight of approximately 120 kg) were used in this study. Six ovaries from six different animals were snap-frozen immediately after slaughter in liquid nitrogen and stored at −80 °C until further immunohistochemical processing. The other 54 ovaries were immediately after slaughter washed in 0.01 M phosphate-buffered saline (PBS) pH 7.4, transferred to a 30 °C PBS solution containing 1% penicillin-streptomycin, and transported to the laboratory within 1 h after slaughter for follicle dissection. To ensure that follicle dissection was finished within a reasonably short time frame, the sows were slaughtered at three different days (three different batches). At each slaughter day, 15 to 20 ovaries were collected for antral follicle dissection. While dissecting one ovary, the remaining ovaries of that batch were kept in Dulbecco’s phosphate buffered saline (DPBS) on ice. For each batch, 4–7 mm in diameter healthy antral follicles and atretic antral follicles were dissected at random within 1.5 h after arrival in the lab. Antral follicle size was estimated by measurement of two perpendicular diameters using a millimeter scale.

Follicles were classified as healthy antral (HA) or atretic antral (AA) follicles based on the presence or absence of blood vessels in the follicle wall and the degree of follicular clarity under a stereomicroscope, as described previously [7,16,17,18]. Briefly, HA follicles were characterized by a vascular sheath on the follicular surface with a pinkish color and clear follicular fluid in the antrum. AA follicles were identified by an opaque color, the absence of recognizable vascularization on the surface of the follicle, and a large number of debris floating in the follicular fluid [7,18,19]. Using the criteria from Gioia et al. [19], these AA follicles were considered to be at an advanced stage of atresia. To further confirm the correctness of this morphological classification, histological analysis using HE staining and immunohistochemical staining with the apoptosis marker cleaved Caspase 3 were performed as described previously [6]. 

Based on the criteria described above, 120 follicles (60 HA follicles and 60 advanced AA follicles) were selected from a pool of 235 follicles; the 115 follicles that were not included in the follicle selection did not meet our selection criteria for healthy and advanced atretic antral follicles and were thus excluded from sampling. The two pools of 60 follicles were randomly divided into six groups consisting of 10 follicles each. Follicular fluid was collected and pooled per 10 follicles. In advanced stage AA follicles, apoptotic granulosa cells had largely become loose from the follicle wall and were floating in the antrum, mixing with the follicular fluid. When dissecting these follicles, the follicular fluid and floating apoptotic granulosa cells were collected together in a Petri dish. The mural granulosa cells of these atretic follicles were scraped from the follicle wall and mixed with the follicular fluid to obtain a complete sample of granulosa cells (floating and mural cells). To create comparable granulosa cell samples, the mural granulosa cells scraped from the walls of healthy antral follicles were also mixed with the follicular fluid (for details see [6]). The purity of the granulosa cell isolates was confirmed as described previously [6]. For each follicle, the granulosa cell follicular fluid solution was diluted 1:3 in PBS, transferred to a 1.5 mL Eppendorf (EP) tube, and centrifuged at 800 g for 5 min. The supernatant was collected and stored at −80 °C for subsequent hormone analysis. The residual granulosa cells remaining in the precipitate were stored at −80 °C until RNA isolation.

### 2.3. Follicle Fluid Testosterone and Progesterone Measurements 

Follicular fluid (FF) estradiol content was measured in our previous study [6]. Testosterone (follicle fluid diluted 1:6; Porcine Testosterone ELISA Kit; cat. no. CSB-E06796p, CUSABIO, Wuhan, China) and progesterone (follicle fluid diluted 1:6; Porcine Progesterone ELISA Kit, cat. no. CSB-E12869p, CUSABIO) contents were determined according to the protocol of the suppliers. The inter-assay variation was less than 10% for all assays. The detection limits for the assays were: 0.1 ng/mL for testosterone and 0.15 ng/mL for progesterone.

### 2.4. RNA Library Construction and Illumina Sequencing 

Total RNA from granulosa cells of three randomly selected samples of pooled granulosa cells from 10 HA and 10 AA follicles, respectively was isolated using TRIzol (Life Technologies, Shanghai, China) for the purpose of RNA-sequencing (RNA-seq). High throughput transcriptome sequencing was carried out by Cloud-Seq Biotech (Shanghai, China). Briefly, total RNA extraction and rRNA depletion were performed using the Ribo-Zero rRNA Removal Kit (Illumina, Shanghai, China) according to the manufacturer’s instructions. RNA libraries were constructed from the rRNA-depleted RNA using the TruSeq Stranded Total RNA Library Prep Kit (Illumina) according to the manufacturer’s instructions. Quality and quantity of the libraries were checked using the BioAnalyzer 2100 system (Agilent Technologies, Palo Alto, CA, USA). Ten pM libraries were denatured, captured on Illumina flow cells, amplified in situ, and finally sequenced for 150 cycles using the Illumina HiSeq 4000 sequencer according to the manufacturer’s instructions. All Illumina sequencing data have been submitted to the Gene Expression Omnibus (GEO) under accession number (GSE136589).

### 2.5. RNA-Seq Data Analysis

Data analysis was performed according to Veno et al. [20] with minor modifications. Briefly, paired-end reads were harvested from the Illumina HiSeq 4000 sequencer; quality control was performed by Q30. After 3′adaptor-trimming, low-quality reads were removed using Cutadapt software (v1.9.3) [21]. The remaining high-quality trimmed reads were used to analyze circRNAs [6], lncRNAs (unpublished data), and mRNA expression profiles.

The high-quality reads were aligned to the pig reference genome (UCSC susScr3) using Hisat2 software [22]. Transcript isoforms were assembled using Cufflinks (v2.2.1) software [23] and combined with the gene annotations from the National Center for Biotechnology Information (NCBI) (ftp://ftp.ncbi.nih.gov/genomes/Sus_scrofa/GFF). The analysis of differential gene and isoform expression was performed with DESeq v1.39.0 [24]. GO and pathway enrichment analyses were performed for the differentially expressed mRNAs using the Database for Annotation, Visualization and Integrated Discovery (DAVID) v6.8 [25]. Gene Set Enrichment Analysis (GSEA v4.02) [26] was used to further confirm the DAVID analysis. For GSEA, all detectable genes were ranked on the basis of a score calculated from the fold change and the adjusted p value. This pre-ranked list was used for analysis by the GSEA Preranked tool following previously described protocols [27].

### 2.6. Quantitative Real-Time RT-PCR

Quantitative Real-Time RT-PCR (qRT-PCR) was used to validate the RNA-Seq data, as described previously [28]. One μg RNA of each sample was used for cDNA synthesis using the PrimeScript RT Master Mix (Takara, Guangzhou, China). Quantitative RT-PCR reactions were performed employing SYBR Premix Ex Taq II (Takara). Individual samples were measured in duplicate. A standard curve using serial dilutions of pooled sample (cDNA from all samples), a negative control without cDNA template, and a negative control without reverse transcriptase (RT) were included in every assay. Only standard curves with efficiency between 90% and 110% and a correlation coefficient above 0.99 were accepted. Data were normalized against the reference gene *Rps18*, which was chosen based on stable gene expression levels (geNorm; Ghent University Hospital, Ghent, Belgium). Primers were designed using the National Center for Biotechnology Information Primer-Blast (http://www.ncbi.nlm.nih.gov/). The primers used and PCR annealing temperatures for each gene are summarized in Table S1.

### 2.7. Immunohistochemistry 

Frozen ovaries (n = 6) were serial sectioned (7 µm). Sections from each of the six ovaries were selected at random and mounted on Superfrost plus glass slides (Menzel-Gläser, Braunschweig, Germany). To determine the presence of proteins (Ki67 and 8-OHdG) in porcine ovaries, immunohistochemistry was performed according to Meng et al. with modifications [12,28]. For each antibody tested, all ovarian sections were stained in one run, in order to be able to compare the immunohistochemical staining among the different sections. Briefly, sections were air-dried for 30 min, and fixed in 4% phosphate buffered paraformaldehyde for 10 min. Slides were subsequently washed in water and microwaved in sub-boiling 0.1 M sodium citrate buffer (pH = 6) for 10 min for epitope antigen retrieval. Slides were cooled down to room temperature and rinsed with Tris-buffered saline (TBS) pH 7.4. Endogenous peroxidase activity was blocked with 3% (*v*/*v*) hydrogen peroxide in methanol. Aldehyde residues were blocked with 0.3% glycine in TBS for 30 min. After rinsing with TBS, sections were incubated with 5% (*wt*/*v*) normal goat serum (Vector Laboratories, Burlingame, CA, USA) in TBS for 60 min at room temperature. Following the removal of the serum, sections were incubated overnight at 4 °C in a humidified chamber with the respective primary antibodies (Ki67, diluted 1:500; 8-OHdG, diluted 1:500) in TBS-BSAc (Aurion, Wageningen, The Netherlands). The sections were rinsed and incubated at room temperature with the corresponding secondary biotin-labeled antibody diluted 1:200 in TBS-BSAc. After rinsing with TBS, the avidin-biotin complex (ABC kit elite, Vector Laboratories) was diluted 1:1000 (*v*/*v*) in TBS-BSAc. Bound antibodies were visualized using 3-3′ diaminobenzidine (Immpact DAB kit, Vector Laboratories) diluted 1:400 (*v*/*v*). The sections were counterstained with Mayer’s haematoxylin. In case of immunofluorescent staining the hydrogen peroxide-methanol step was left out. Briefly, after primary antibody incubation (aromatase, diluted 1:200; GCLC, diluted 1:200 all in TBS-BSAc, overnight at 4 °C), sections were rinsed with TBS and incubated in the dark with a secondary Alexa fluor 488 labeled goat-anti-rabbit antibody (A-11008, ThermoFisher Scientific, Waltham, MA, USA) diluted 1:200 (*v*/*v*) in TBS-BSA-c for 1 h at room temperature. Sections were counterstained with DAPI (0.5 μg/mL; Sigma) for 10 min. Sections were imaged at 20 times magnification using a fluorescence microscope (Leica DM6B), a digital camera (DFC365 FX), and imaging software (LasX; all Leica Microsystems, Amsterdam, The Netherlands). The mean staining intensity of the granulosa cell layer was determined using ImageJ (NIH, Bethesda, MD, USA). Control sections were incubated with isotype IgG (Vector Laboratories), instead of the respective primary antibodies, according to the manufacturer’s instructions. Background staining in these controls was negligible.

### 2.8. Statistical Analysis

GraphPad Prism version 7.00 (Graphpad Software, San Diego, CA, USA) was used for statistical analysis. Data were expressed as mean ± standard error of the mean (SEM). Data was checked for normality and when normality was confirmed the Student’s *t* test was used for data analysis. If normality could not be assumed, data were log10 transformed. If data was still not normally distributed a Mann–Whitney U non-parametric test was applied. *p* values < 0.05 were considered to be significantly different.

## 3. Results

### 3.1. Differentially Expressed Genes during Follicular Atresia

**Figure 1:**
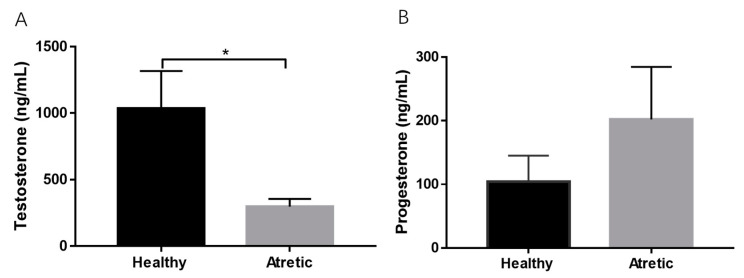
Hormone concentrations in follicular fluid of healthy antral (HA) and antral atretic (AA) follicles (4–7 mm). (**A**) Testosterone concentrations (ng/mL). (**B**) Progesterone concentrations (ng/mL). Follicular fluid from pooled samples (n = 4). Values represent means +/− SD. *, *p* < 0.05.

Testosterone concentrations were significantly decreased in AA FF compared with HA FF, while no difference was observed in progesterone content between HA and AA FF (Figure 1A,B).

**Figure 2:**
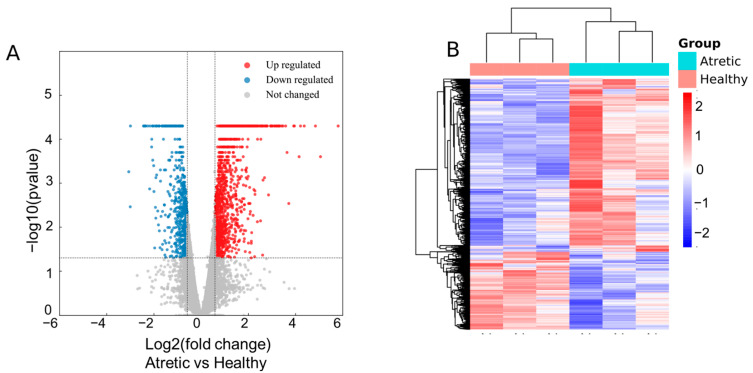
Expression profiles of differentially expressed genes (DEGs). (**A**) Volcano plot visualizing the statistical difference of the DEGs. The horizontal axis represents the fold-change (FC) of detected transcripts and the vertical axis represents the *p*-value. The red dots in plot denote the significantly different DEGs (FC ≥ 1.5 and *p* ≤ 0.05, respectively); (**B**) Hierarchical clustering showing the expression profiles of all DEGs. Rows represent DEGs while columns represent different samples. The DEGs were classified according to Pearson correlation analysis.

To reveal the normal transcriptomic changes that occur between HA and AA follicles, RNA-seq was conducted on the RNA isolated from granulosa cells. There were in total around 15,205 mRNA transcripts detected in HA and AA follicles, of which 2160 were differentially expressed genes (DEGs) (absolute fold change ≥1.5 and *p* ≤ 0.05). Among these DEGs, 1971 transcripts were annotated transcripts while 189 were unknown transcripts. The 2160 DEGs included 677 downregulated transcripts and 1483 significantly upregulated transcripts (Figure 2A). All up-regulated and down-regulated mRNA transcripts were further evaluated by unsupervised hierarchical clustering analysis (Figure 2B). This analysis showed a good correlation within groups and a clear distinction between HA and AA follicles.

### 3.2. Transcripts with Greatest Fold Differences 

A list of 50 top transcripts more highly expressed in granulosa cells of atretic antral follicles versus healthy antral follicles is shown in Table 1. *CXCL13*, encoding the chemokine C-X-C motif ligand 13, an inflammation factor, heads the list followed by two unknown transcripts ENSSSCG00000001783 and ENSSSCG00000026265. Several other genes directly related to the process of inflammation including CHI3L1 (encoding a glycoprotein member of the glycosyl hydrolase 18 family), members of the Toll-like receptor family TLR4 and TLR9, and CCR1 (C-C Motif Chemokine Receptor 1), encoding a member of the beta chemokine receptor family, were all highly expressed in atretic antral follicles. Moreover, the pro-apoptotic gene *TGFβ2*, encoding the secreted ligand transforming growth factor beta-2 (TGFβ-2), together with *TGFβ* receptor 2 (*TGFBR2*) were also much more highly expressed by granulosa cells of AA compared to HA follicles.

In contrast, steroid hormone biosynthesis related genes including steroidogenic acute regulatory protein (StAR) and luteinizing hormone chorion gonadotropin receptor (LHCGR) were included in the list of top 50 transcripts most highly expressed in granulosa cells of HA follicles compared to AA follicles (Table 2). 

### 3.3. Bioinformatic Analysis of RNA-Seq Data 

**Figure 3:**
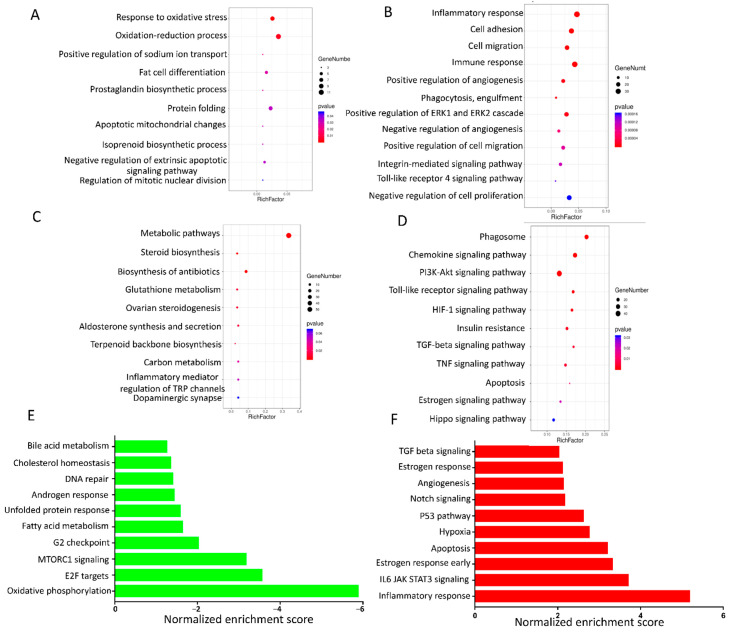
GO and pathway analysis. (**A**) Biological process for the downregulated DEGs; (**B**) Biological process for the upregulated DEGs; (**C**) KEGG pathway for the downregulated DEGs; (**D**) KEGG pathway for the upregulated DEGs; The size and color of each bubble represents the number of genes in each pathway and P value, respectively. (**E**) Down-regulated pathways and (**F**) up-regulated pathways by GSEA using the pre-ranked list of all expressed genes and compared to the GSEA database Hallmark gene sets.

Using DAVID software, the GO and KEGG pathway were analyzed for downregulated and upregulated genes in AA follicles compared with HA follicles. For the downregulated genes, GO analysis showed that the top significantly enriched biological processes were “response to oxidative stress”, “oxidation-reduction process”, and “regulation of mitotic nuclear division” (Figure 3A). In line with these results, KEGG analysis showed that the downregulated genes were markedly enriched for “metabolic pathways”, and the oxidative stress related pathway “Glutathione metabolism” (Figure 3B). Additionally, “Steroid biosynthesis” and “Ovarian steroidogenesis” pathways were also significantly enriched, belonging to the top enriched pathways (Figure 3B).

For the upregulated genes, GO analysis showed that the top significantly enriched pathways were inflammatory response related including “Inflammatory response”, “Immune response”, “Phagocytosis, Engulfment”, “Integrin-mediated signaling pathway”, and “Toll-like receptor 4 signaling pathway” (Figure 3C). In support of these results, KEGG analysis showed that upregulated genes were also enriched in the inflammation related pathway, including “Phagosome”, “Chemokine signaling pathway” and “Toll-like receptor signaling pathway” (Figure 3D). Next to pathways related to inflammation, apoptosis related pathways including “HIF-1 signaling pathway”, “TGF-beta signaling pathway”, “TNF signaling pathway”, and “Apoptosis” were also significantly enriched (Figure 3D).

Our DAVID analysis results were further supported by GSEA, which was used to characterize the RNA-Seq data by comparing the pre-ranked list of all expressed genes in our experiment to the GSEA database gene sets. GO and KEGG gene sets also revealed a significant enrichment in downregulated genes involved in oxidative stress and steroid hormone biosynthesis, and upregulated genes involved in inflammation and apoptosis processes in the granulosa cells of AA follicles (Figure S1). The GSEA Hallmark gene sets further revealed that the downregulated genes were significantly enriched in the pathways including “Oxidative phosphorylation”, cell cycle related “G2 checkpoint”, and “Cholesterol homeostasis” (Figure 3E) while the upregulated genes were strongly enriched in pathways such as Inflammation response” and apoptosis related gene sets including “Apoptosis”, “Hypoxia”, “P53 pathway” and “TGFB signaling” (Figure 3F). 

Based on the above extensive molecular pathway analysis results, the two top regulated processes “ovarian steroidogenesis” and “responsive to oxidative stress” were selected for further functional analysis, as these two processes exert direct roles in ovarian follicular development and atresia.

### 3.4. Downregulated Genes Involved in the Ovarian Steroidogenesis

**Figure 4:**
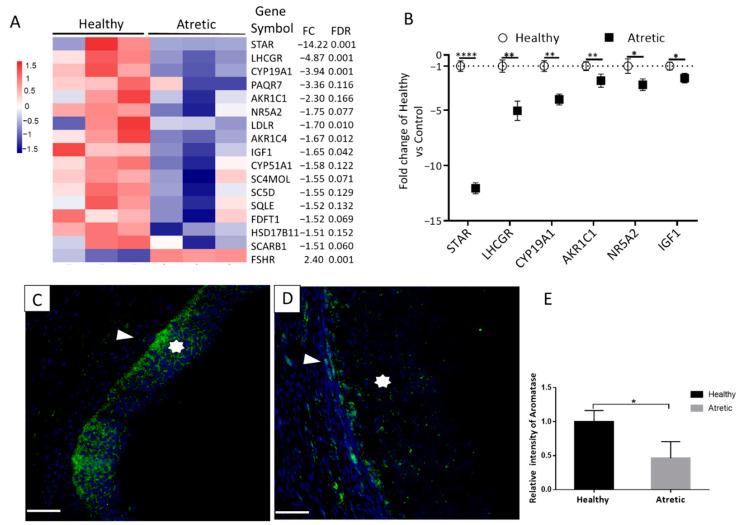
Significantly downregulated gene profiles involved in ovarian steroidogenesis. (**A**) The significantly downregulated genes were shown in the heatmap (n = 3), FDR, false discovery rate and fold change (atretic over healthy). (**B**) Validation of selected downregulated genes using qRT-PCR. Gene expression as fold change of atretic over healthy follicles (n = 6), with no change indicated as 1. Healthy: open circles, Atretic: filled squares. Representative aromatase immunostaining in healthy (**C**) and atretic antral follicles (**D**). Scale bars represent 50 µm; Asterisk indicates granulosa cells. (**E**) Quantification of aromatase fluorescence intensity (n = 6 different animals) showed significantly decreased intensity in the granulosa cells in atretic follicles compared with healthy follicles. Data as mean ± SD. Analysis with Students *t*-test. *, *p* < 0.05; **, *p* < 0.01, ****, *p* < 0.0001.

Individual transcript analysis showed a decreased expression of genes involved in ovarian steroidogenesis in AA follicles, e.g., *StAR* (FC = −14.22, FDR = 0.001), *LHCGR* (FC = −4.87, FDR = 0.001), *CYP19A1* (FC = −3.94, FDR = 0.001), AKR1C1 (FC = −2.30, FDR = 0.166), *AKR1C4* (FC = −1.70, FDR = 0.010), *NR5A2* (FC = −2.09, FDR = 0.001), *CYP51A1* (FC = −1.58, FDR = 0.122), and *HSD17B11* (FC = −1.51, FDR = 0.152) (Figure 4A). In addition, granulosa cells of AA follicles had lower *IGF1* expression (FC = −1.65, FDR = 0.042). The mRNA content of the *FSHR* gene however was significantly higher in the granulosa cells of AA follicles compared to HA follicles (see Supplemental Materials Table S1 for details). For technical validation, *StAR*, *LHCGR*, *CYP19A1*, *AKR1C1*, *NR5A2*, *HSD17B11*, and *IGF1* were selected and examined by qRT-PCR (Figure 4B); all these genes were significantly regulated, confirming the RNA-seq data. CYP19A1, 2 and 3, >99% homologous isoforms of aromatase, are mainly expressed in the porcine ovary, placenta and embryonic tissues, respectively [29]. As *CYP19A1* was downregulated, we additionally analyzed aromatase (CYP19A1-3) protein expression. Consistent with mRNA levels, granulosa cells in AA follicles had lower aromatase protein expression compared to HA follicles (Figure 4C–E).

### 3.5. Oxidative Stress and Antral Follicular Atresia

**Figure 5:**
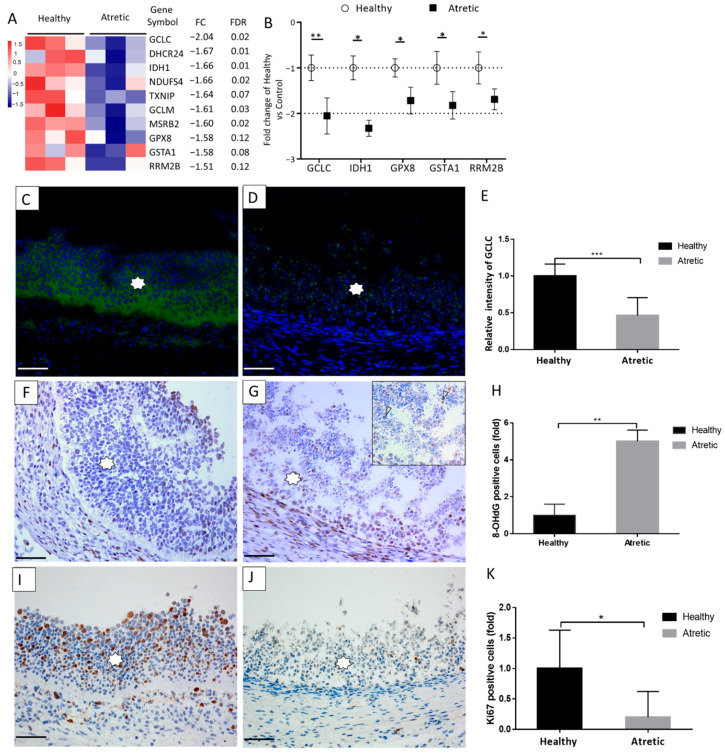
Oxidative stress involved in antral follicular atresia. (**A**) The significantly downregulated antioxidant genes shown in the heatmap (n = 3), FDR, false discovery rate and fold change (atretic over healthy). (**B**) Validation of selected downregulated antioxidant genes using qRT-PCR. Gene expression as fold change of atretic over healthy follicles (n = 6), with no change indicated as 1. Healthy: open circles, atretic: filled squares. (**C**–**K**) Immunostaining for the presence of GCLC, 8-OHdG, and Ki67 (brown staining) as markers of oxidative stress and cell proliferation in healthy (**C**,**F**,**I**) and atretic (**D**,**G**,**J**) follicles (n = 6, different animals). Representative immunofluorescence staining of GCLC (**C**,**D**), DAB staining for the presence of 8-OHdG (**F**,**G**) and Ki67 (**I**,**J**). Immunofluorescence analysis showed the significantly decreased the fluorescent intensity of GCLC in the granulosa cells of AA follicles compared with HA follicles (**E**); The percentage of 8-OHdG immunostaining positive cells in the granulosa cells of AA follicles was much higher than that of HA follicles. On the contrary, the percentage of Ki67 immunostaining positive granulosa cells was significantly decreased in AA follicles compared to HA follicles (**J**). Scale bars represent 50 µm. Insert show a detail of the granulosa layer. Asterisk indicates granulosa cells. Analysis with Students *t*-test. *, *p* < 0.05; **, *p* < 0.01. ***, *p* < 0.001. HA, healthy antral follicles; AA, atretic antral follicles.

Next to genes involved in the ovarian steroidogenesis, individual transcript analysis showed the downregulated expression of genes related to antioxidant processes in the granulosa cells of AA follicles e.g., *GCLC* (FC = −2.04, FDR = 0.02), *DHCR24* (FC = −1.67, FDR = 0.01), *IDH1* (FC = −1.66, FDR = 0.01), *TXNIP* (FC = −1.64, FDR = 0.07), *GCLM* (FC = −1.61, FDR = 0.03), *MSRB2* (FC = −1.60, FDR = 0.02), *GPX8* (FC = −1.58, FDR = 0.12), *GSTA1* (FC = −1.58, FDR = 0.08), and *RRM2B* (FC = −1.51, FDR = 0.12) (Figure 5A). For technical validation, *GCLC*, *IDH1*, *GCLM*, *GPX8*, *GSTA1*, and *RRM2B*, involved in the glutathione metabolism pathway, were selected and analyzed by qRT-PCR (Figure 5B). In line with the RNA-seq data, all these genes were significantly downregulated. We also analyzed the protein expression levels of GCLC, one of the subunits of glutamate-cysteine ligase, which acts as rate-limiting enzyme in glutathione synthesis, and observed in accordance with the mRNA concentration, that granulosa cells in AA follicles had lower GCLC protein expression compared with HA follicles (Figure 5C–E). 

In order to investigate whether the decrease in follicular antioxidant gene and protein expression in granulosa cells of AA follicles coincided with increased oxidative stress, the presence of 8-OHdG, a marker for DNA oxidation during oxidative stress [11], was investigated. The percentage of positive 8-OHdG immunohistochemical staining in apoptotic granulosa cells of AA was more than five times higher than staining in HA follicles (Figure 5F–H), indicative of the presence of oxidative stress in the AA follicles. In line with these observations, the percentage of Ki67-positive granulosa cells in AA follicles was significantly decreased, indicative of reduced proliferation of granulosa cells in AA follicles compared to HA follicles (Figure 5I–K). 

## 4. Discussion

In this study, comprehensive transcriptome gene expression profiling by RNA-Seq analysis was performed to identify DEGs in granulosa cells from HA and AA follicles. Bioinformatics analysis revealed that the downregulated transcripts were mainly highlighted in the steroid biosynthesis pathway and response to oxidative stress processes including antioxidant genes involved in the glutathione metabolism pathway and other redox-related genes. These observations were confirmed by RT-qPCR and immunohistochemistry. Previous studies have characterized the porcine granulosa cells gene expression profiles by means of microarray either in small antral follicles (1–2 mm in diameter) [15] or medium-sized antral follicles (3–5 mm in diameter) during relatively early stages of atresia [16]. The present study is to the best of our knowledge the first RNA-Seq analysis investigating the underlying molecular difference between porcine granulosa cells of HA and advanced AA medium to large-sized (4–7 mm in diameter) follicles.

There are around 2160 DEGs detected that meet the cutoff criteria (FC ≥ 1.5 and *p* ≤ 0.05). When applying for more stringent cutoff criteria (FC ≥ 2.0 and *p* ≤ 0.05), the number of total DEGs is decreased to 1217, which is still more than twice the number of DEGs observed in the study by Zhang et al. [16], who analyzed granulosa cells of medium-sized (3–5 mm) HA and early AA follicles by microarray. The larger number of DEGs detected in our study is most likely explained by the technical advantages of RNA-Seq including its large dynamic detection range of transcripts expression levels and its more quantitative characteristics without sophisticated normalization of data sets [30], as well as by differences in size and developmental stages of the selected antral follicles.

Estradiol is essential for follicular growth and development. In pigs, granulosa cell CYP19A1 expression increases from the start of the follicular phase until the preovulatory stage approximately five days later. When antral follicles are selected into the growing pool of follicles, follicular fluid β-estradiol concentrations increase with follicular diameter [31]. Consistently, our previous research showed a decrease in estradiol levels in AA follicles compared to the HA follicles [6]. Transcriptome analysis reveals the decreased expression of genes involved in pathways responsible for cholesterol and steroid biosynthesis in AA follicles. Immunostaining with an antibody against aromatase (CYP19A1-3) confirms the decreased aromatase expression in granulosa cells of AA follicles at the protein level, which may explain the lower FF estradiol levels in AA follicles.

Additionally, the mRNA content of other steroidogenic enzymes expressed in granulosa cells is significantly downregulated in the AA follicles; e.g., steroidogenic acute regulatory protein (*StAR*), facilitating the cholesterol transfer along the mitochondrial membranes, aldo-keto reductase family 1 member C1 (*AKR1C1*), aldo-keto reductase family 1 member C4 (*AKR1C4*), and hydroxysteroid 17-beta dehydrogenase 11 (*HSD17B11*), involved in the conversion of cholesterol into androgens (reviewed in [32]). The downregulation of *StAR*, *AKR1C1*, *AKR1C4*, and *HSD17B11* may contribute to the decreased testosterone concentrations in the FF of AA follicles compared with HA follicles, which may also further explain the reduced estradiol concentrations in the FF of AA follicles, testosterone being one of the main precursors for estradiol synthesis [33].

Besides genes encoding enzymes participating in steroid metabolism, granulosa cells from HA and AA follicles show a differential expression of genes involved in regulation of enzymes participating in ovarian steroidogenesis. Examples of these differentially expressed genes are *FSHR*, *LHCGR,* and *IGF1*. In rodents, it was originally hypothesized that increased FSHR expression may facilitate antral follicles selection for dominance by augmenting the FSH response, stimulating the production of estradiol [2]. In accordance with this hypothesis lower expression of *FSHR* is reported in early AA small to medium-sized (3–5 mm) follicles [16]. Quite to our surprise, *FSHR* gene expression in porcine granulosa cells of advanced medium to large-sized AA follicles is increased compared with HA follicles. An explanation of this discrepancy may be related to the follicle status (advanced vs early atresia) and follicle size difference (small to medium-sized versus medium to large-sized). Medium to large-sized follicles, in an initial attempt to protect themselves against or survive its atretic fate, may respond by increasing FSHR expression with subsequent FSH binding providing an antiapoptotic signal. At later stages of follicular development activation of the FSH signaling pathway is apparently not enough to maintain healthy follicular development, thus facilitating follicular atresia. In support of this assumption, Liu et al. demonstrated that in healthy medium to large sized antral porcine follicles FSHR expression is lower in granulosa cells compared to granulosa cells of smaller follicles [34]. This implies that in order for the granulosa cells to survive, LH signaling may also be needed. Indeed, porcine follicles of around 4–5 mm in diameter start to express LHCGR in the granulosa cells layer, possibly fostering the shift from FSH to LH dependence [35]. Other studies show that LH can activate the aromatase enzyme and thus facilitating estradiol production via the activation of the LHCGR in human luteinized granulosa cells [36]. It therefore does not come as a surprise that the loss of LHCGR mRNA expression in porcine granulosa cells is associated with advanced AA of medium to large-sized follicles. Granulosa cells in HA follicles further show a higher expression of the *IGF1* gene than granulosa cells in advanced AA follicles, which could explain the higher IGF1 concentrations in FF of HA follicles [6]. IGF1 enhances the effects of LH on steroidogenesis including estradiol synthesis in porcine granulosa cells [37,38]. Together, FSH, LH, IGF1, and estradiol have been implicated as anti-apoptotic factors [39,40]; lack of these factors could lead to granulosa cells apoptosis, a process in which oxidative stress may play a role.

Transcriptome analysis further revealed a decreased expression of genes involved in the glutathione metabolism pathway in granulosa cells of AA follicles. For example, five of these transcripts (*GCLC*, *GCLM*, *IDH1*, *GSTA1*, and *GPX8*) are significantly downregulated in advanced AA follicles. GCLC and GCLM are two subunits of glutamate-cysteine ligase, which acts as a rate-limiting enzyme in glutathione (GSH) synthesis, an important antioxidant to detoxify H_2_O_2_ [41]. The decreased mRNA content of *GCLC* in AA follicles is confirmed by immunofluorescence staining, supporting the hypothesis that ROS levels may be increased in these granulosa cells. This assumption is further supported by the decreased mRNA content of *IDH1* in AA follicles. IDH1 is an important generator of NADPH in cells, essential for the regeneration of GSH from GSSH [42]. By means of single cell sequencing, the analysis of cell-type-specific aging-associated transcriptional changes uncovered that granulosa cells in the aged non-human primate ovary prone to higher oxidative stress, had a lower *IDH1* expression compared with the younger controls. Further functional studies using the human KGN granulosa cell line show that silencing of IDH1 leads to increased ROS levels, inducing granulosa cell apoptosis [11]. Next to genes related to GSH production, support for the assumed increase in ROS levels in apoptotic granulosa cells comes from decreased expression of DEGs that use GSH to detoxify increased ROS levels. Examples of these genes are GSTA1 which function is to expose GSH to target electrophilic compounds, including products of oxidative stress, for detoxification. Similarly, GPx8, a new member of the glutathione peroxidase (GPx) family (GPx 1-8), uses GSH to reduce H_2_O_2_ and other ROOH [43]. Although less studied in the ovary, it is reported that GPx8 overexpression can significantly improve endoplasmic reticulum (ER) antioxidative capacity induced by lipotoxicity in INS-1E β-cells [44]. These results direct to a presumptive role for reduced glutathione metabolism pathway activity in antral follicular atresia.

The assumption that antioxidant capacity in granulosa cells of AA follicles is decreased, is further supported by the downregulation of other redox-related genes including *RRM2B*, *NDUFS4*, *DHCR24,* and *MSRB2*. RRM2B, a stress response protein, has been reported to protect human fibroblast cells from oxidative stress by decreasing ROS levels [45]. NDUFS4 encodes an accessory subunit of the first mitochondrial oxidative phosphorylation complex; increased ROS levels and aberrant mitochondrial morphology are observed in primary muscle cells and skin fibroblasts from *NDUFS4*^−/−^ mice [46]. In vitro experiments in MIN6 cells implicate that DHCR24 (encoding 3β-Hydroxysteroid-Δ24 Reductase) inhibits the generation of intracellular ROS protecting these cells from apoptosis. Finally, MSRB2 (encoding methionine sulfoxide reductase B2) catalyzes the reduction of free and protein-based methionine sulfoxides to methionine. MSRB2 protects against cell damage by mitochondria-related oxidative stress in several tissues [47]. The decreased expression of these genes in granulosa cells from AA follicles points towards a role for oxidative damage in follicular atresia. This presumption is supported by immunohistochemical staining for 8-OHdG. 8-OHdG is a biomarker for oxidative stress, one of the predominant forms of free radical-induced oxidative lesions located in nuclear and mitochondrial DNA [48]. Moderate to strong 8-OHdG nuclear immunostaining is observed in granulosa cells of AA follicles while staining is faint to absent in granulosa cells of HA follicles.

In correspondence with the above, the apoptosis related pathways including “HIF-1 signaling pathway”, “TGF-beta signaling pathway”, “TNF signaling pathway”, “p53 pathway”, and “Apoptosis” predominated when performing pathway analysis for up-regulated genes in advanced AA follicles. TGFβ [49], TNFα [50] and p53 signaling [6] have been indicated to be involved in ovarian follicular atresia. Taking TGFβ as an example [6], differentially expressed genes involved in the TGFβ signaling pathway are *TGFβ1*, *TGFβ2*, *SMAD2*, and *SMAD7*. In vitro studies have shown that independent of the presence of FSH, the addition of TGFβ1 to the culture medium promotes the apoptosis of bovine granulosa cells accompanied by a decrease in proliferation [51], which is in line with our observed decrease in Ki67 immunostaining in granulosa cells of AA follicles. Similarly, in the human KGN granulosa cell line, the supplementation of TGFβ2 increases the incidence of apoptosis [52]. TGFβ promotes granulosa cell apoptosis by affecting Smad7 expression as overexpression of Smad7 markedly increased apoptosis in mouse granulosa cells, while reduced expression of Smad7 significantly blocks TGFβ induced apoptosis [53]. Moreover, knockdown of Smad2 in human HK2 cells alleviated p53-mediated cell apoptosis [54].

## 5. Conclusions

In conclusion, our study, by means of RNA-Seq analysis, provided a comprehensive transcriptome difference between HA and advanced AA follicles. The results of this study showed that the decrease in the expression of genes related to steroid hormone synthesis and anti-apoptotic factors coincides with decreased the expression of genes involved in redox signaling pathways leading to an increased expression of oxidative stress markers, all presumably contributing to antral follicular atresia.

## Figures and Tables

**Table 1:** Top 50 of the most highly expressed genes (FDR ≤ 0.001) between atretic (*n* = 3) vs. healthy (*n* = 3) antral follicles.

Gene_ID	Gene Symbol	Fold Change	Gene_ID	Gene Symbol	Fold Change
ENSSSCG00000028731	CXCL13	86.58	ENSSSCG00000010816	TGFB2	21.35
ENSSSCG00000001783	-	85.80	ENSSSCG00000004484	-	21.13
ENSSSCG00000026265	-	64.97	ENSSSCG00000022896	-	20.99
ENSSSCG00000027023	CEMIP	54.99	ENSSSCG00000006243	PENK	20.70
ENSSSCG00000012113	AMELX	54.01	ENSSSCG00000009280	-	19.92
ENSSSCG00000005311	CD72	38.09	ENSSSCG00000025058	NRAMP1	19.32
ENSSSCG00000008995	LRAT	37.74	ENSSSCG00000022741	LOC102166564	18.91
ENSSSCG00000010624	DUSP5	37.12	ENSSSCG00000014442	PDGFRB	18.52
ENSSSCG00000018032	TRPV2	36.11	ENSSSCG00000017956	CD68	18.36
ENSSSCG00000008937	AMBN	35.66	ENSSSCG00000015476	CHI3L1	18.18
ENSSSCG00000010504	BLNK	34.22	ENSSSCG00000015850	DUSP4	16.87
ENSSSCG00000029163	BCAT1	32.99	ENSSSCG00000020967	-	16.83
ENSSSCG00000023102	CACNG4	31.51	ENSSSCG00000022236	FOLR1	16.38
ENSSSCG00000024134	MGLL	30.61	ENSSSCG00000011534	BHLHE40	16.36
ENSSSCG00000010383	WDFY4	30.40	ENSSSCG00000016830	PRLR	16.27
ENSSSCG00000027643	DIO2	30.38	ENSSSCG00000016855	FYB	16.08
ENSSSCG00000004053	TAGAP	29.82	ENSSSCG00000011457	IL17RB	15.78
ENSSSCG00000006472	CRABP2	28.48	ENSSSCG00000001457	SLA-DQB1	15.71
ENSSSCG00000025551	RUNX2	28.00	ENSSSCG00000011322	CCR1	15.45
ENSSSCG00000008348	PLEK	26.96	ENSSSCG00000027607	IER3	15.02
ENSSSCG00000005503	TLR4	26.71	ENSSSCG00000003524	C1QA	14.80
ENSSSCG00000009455	-	24.76	ENSSSCG00000000687	CD4	14.33
ENSSSCG00000030275	COL12A1	22.88	ENSSSCG00000013788	-	14.32
ENSSSCG00000011226	TGFBR2	22.74	ENSSSCG00000003465	FBLIM1	14.19
ENSSSCG00000011436	TLR9	22.51	ENSSSCG00000008128	DUSP2	14.19

For each gene, the fold-change (FC) of atretic antral follicles vs. healthy antral follicles is shown.

**Table 2:** Top 50 of most lowly expressed genes (FDR ≤ 0.001) between atretic (*n* = 3) vs. healthy (*n* = 3) antral follicles.

Gene_ID	Gene Symbol	Fold Change	Gene_ID	Gene Symbol	Fold Change
ENSSSCG00000005577	LOC100156463	−36.96	ENSSSCG00000028695	TMSB15A	−5.20
ENSSSCG00000022703	MRPL27	−19.76	ENSSSCG00000029587	TIMP4	−5.15
ENSSSCG00000026109	STAR	−14.22	ENSSSCG00000021652	LOC100515066	−5.14
ENSSSCG00000021572	-	−13.66	ENSSSCG00000028603	-	−5.14
ENSSSCG00000027253	HIST1H4B	−9.60	ENSSSCG00000003848	APOER2	−5.12
ENSSSCG00000004755	DLL4	−8.01	ENSSSCG00000004360	-	−5.11
ENSSSCG00000024428	CHRNA9	−7.95	ENSSSCG00000009153	DKK2	−5.06
ENSSSCG00000004413	PPIL6	−7.80	ENSSSCG00000000795	ADAMTS20	−5.03
ENSSSCG00000026801	LOC100738273	−7.62	ENSSSCG00000023100	-	−4.96
ENSSSCG00000025691	BCL10	−7.44	ENSSSCG00000005221	SPATA6L	−4.94
ENSSSCG00000023023	LOC106504822	−7.28	ENSSSCG00000009406	HTR2A	−4.92
ENSSSCG00000004754	CHAC1	−7.23	ENSSSCG00000008421	LHCGR	−4.87
ENSSSCG00000025667	FBXO2	−6.98	ENSSSCG00000022073	ZBTB38	−4.69
ENSSSCG00000022061	TRMT12	−6.95	ENSSSCG00000009069	C4orf33	−4.64
ENSSSCG00000012005	-	−6.58	ENSSSCG00000010344	NRG3	−4.58
ENSSSCG00000026035	LOC100514471	−6.52	ENSSSCG00000005276	RFK	−4.38
ENSSSCG00000029066	IDI1	−6.37	ENSSSCG00000004263	ZUFSP	−4.31
ENSSSCG00000004601	MNS1	−6.07	ENSSSCG00000010485	-	−4.31
ENSSSCG00000016086	-	−5.93	ENSSSCG00000000475	IRAK3	−4.22
ENSSSCG00000001942	SLC25A21	−5.92	ENSSSCG00000027128	LOC100622930	−4.21
ENSSSCG00000027704	LOC100739098	−5.73	ENSSSCG00000027422	LOC100623180	−4.20
ENSSSCG00000028476	LOC100511313	−5.60	ENSSSCG00000006987	SLC7A2	−4.19
ENSSSCG00000014314	-	−5.57	ENSSSCG00000016958	PIK3R1	−4.10
ENSSSCG00000023260	-	−5.36	ENSSSCG00000015870	KCNJ3	−4.06
ENSSSCG00000026079	TKTL2	−5.35	ENSSSCG00000002650	APRT	−4.02

For each gene, the fold-change (FC) of atretic antral follicles vs. healthy antral follicles is shown.

## Data Availability

All datasets generated for this study are included in the article/Supplementary Materials.

## Supplementary Materials

The following are available online at https://www.mdpi.com/2076-3921/10/1/22/s1, Figure S1 Representative result of GSEA. A pre-ranked list of all expressed genes (nonredundant human gene symbols) was used for GSEA and compared to gene sets of the GSEA gene set database C5: GO biological process gene sets and C2: KEGG gene sets. Significantly enriched GO biological process in the response to oxidative stress (A), inflammation response (B); Significantly enriched KEGG pathways in steroid hormone biosynthesis (C), apoptosis (D). Table S1: Sequences of the primers used for qRT-PCR.
