# Supplementary material for: Transcriptome Analysis of Porcine Granulosa Cells in Healthy and Atretic Follicles: Role of Steroidogenesis and Oxidative Stress
Source: Antioxidants (Basel). 2020 Dec 28;10(1):22. doi: 10.3390/antiox10010022 (PMC7824097; doi:10.3390/antiox10010022)
Supplement: Supplementary file 1 [file antioxidants-10-00022-s001.pdf]

Table S1. Sequences of the primers used for qRT-PCR.

Circle Gene Symbol		Primers (5'-3')	Anealing Temp (°C)	Length of Product
STAR	Forward	GTGGAACCCCAGTGTCAAGA	60.00	129
	Reverse	CAGCGCACACTCACAAAGTC		
LHCGR	Forward	AACCACCGTACCAGCAAATG	59.00	136
	Reverse	CTCCTTCAGCTCCAGGGAAA		
CYP19A1	Forward	GATCCCACTGGACGAAAAGGC	60.00	117
	Reverse	TACTTTCTGTACAGCCAAGGAATC		
AKR1C1	Forward	GCACCTGAAGAGGTTCCCAA	60.00	133
	Reverse	TCTTGCTTCGAATGGCCTGT		
NR5A2	Forward	CGCCAATGGTTTCTACTTTAAGC	59.00	186
	Reverse	AAATCGACAGTAAGGACAGAGC		
HSD17B11	Forward	GGATGCATAGTCCAGGAATGC	60.00	127
	Reverse	TGTCTGCAGTCCAGTTGCTT		
IGF1	Forward	TGCTTGCTCTCCTTCACCAG	60.00	124
	Reverse	ACCCTGTGGGCTTGTTGAAA		
GCLC	Forward	GATCCTCCAGTTCCTGCACA	60.00	87
	Reverse	GAGAGAGAACCAACCTCGTCG		
GCLM	Forward	ACCAGTGGGCACAGGTAAAA	60.00	200
	Reverse	CCACTCATGTGCCTCGATGT		
IDH1	Forward	TCTGTGGCCCAAGGTTATGG	60.00	149
	Reverse	GGATTGGTGGACGTCTCCTG		
GPX8	Forward	TGCAGTTTTGCTGTCTATGGT	60.00	167
	Reverse	GGTTCCTTCTTTGAAGAATTTTGCC		
GSTA1	Forward	CTCTGCTGAAGGCCCTGAA	60.00	179
	Reverse	TCAGAAGATTGGTCCTGGGTG		
RRM2B	Forward	GGTCTGAACAGGAGGAGTTAGG	60.00	100
	Reverse	AGTGGTGTTTGCTTAGACGC		
RPS18*	Forward	ATTGCCTTTGCTATCACTGCG	60.00	132
	Reverse	GGTGATTACACGTTCCACCTCA		

Gene denoted with an asterisk was used as reference gene for normalization.Abbreviations: *STAR*, steroidogenic acute regulatory protein; *LHCGR*, luteinizing hormone/choriogonadotropin receptor; *CYP19A1*, cytochrome P450 19A1; *AKR1C1*, aldo-keto reductase family 1, member C1; NR5A2, nuclear receptor subfamily 5 group A member 2; *HSD17B11*, estradiol 17-beta-dehydrogenase 11; *IGF1*, insulin like growth factor 1; *GCLC*, glutamate-cysteine ligase catalytic subunit; *GCLM*, glutamate-cysteine ligase modifier subunit; *IDH1*, isocitrate dehydrogenase (NADP(+)) 1; *GPX8*, glutathione peroxidase 8; *GSTA1*, glutathione S-transferase alpha 1; *RRM2B*, ribonucleotide reductase regulatory TP53 inducible subunit M2B; *RPS18*, ribosomal protein S18.

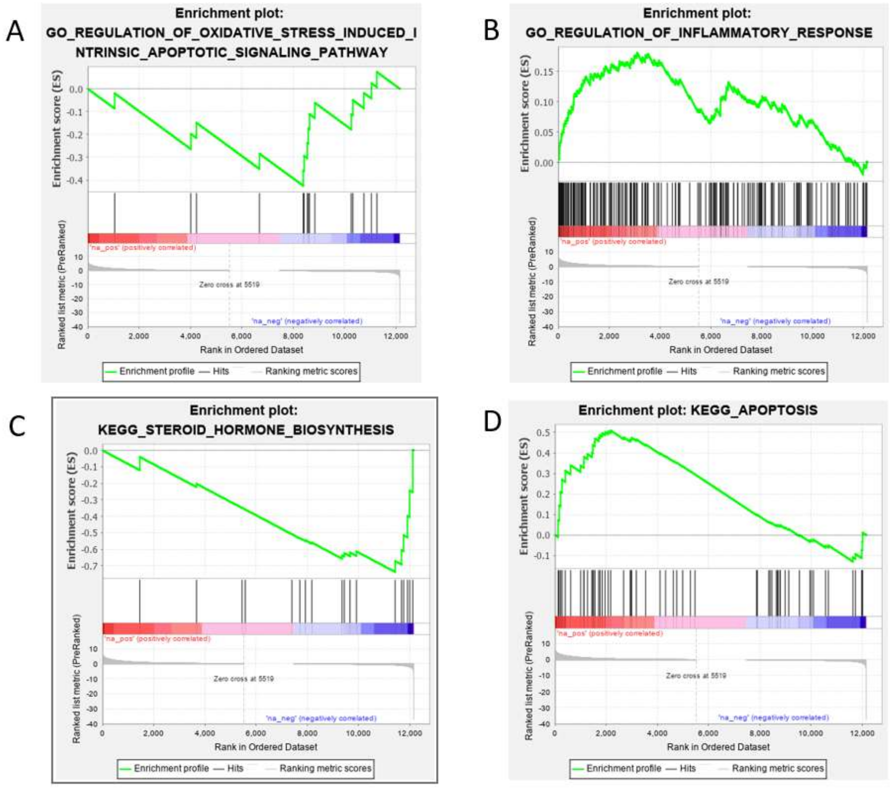

Figure S1. Representative result of GSEA. A pre-ranked list of all expressed genes (nonredundant human gene symbols) was used for GSEA and compared to gene sets of the GSEA gene set database C5: GO biological process gene sets and C2: KEGG gene sets. Significantly enriched GO biological process in the response to oxidative stress (**A**), inflammation response (**B**); Significantly enriched KEGG pathways in steroid hormone biosynthesis (**C**), apoptosis (**D**).
